# Supplementary material for: Plasma-Derived Extracellular Vesicles Convey Protein Signatures That Reflect Pathophysiology in Lung and Pancreatic Adenocarcinomas
Source: Cancers (Basel). 2020 May 2;12(5):1147. doi: 10.3390/cancers12051147 (PMC7281335; doi:10.3390/cancers12051147)
Supplement: Supplementary file 1 [file cancers-12-01147-s001.zip › cancers-781548-suppl-XML/cancers-781548-Supplementary Figures 1-3.pdf]

Supplementary Materials: Plasma-Derived Extracellular Vesicles Convey Protein Signatures that Reflect Pathophysiology in Lung and Pancreatic Adenocarcinomas

Johannes F. Fahrmann, Xiangying Mao, Ehsan Irajizad, Hiroyuki Katayama, Michela Capello, Ichidai Tanaka, Taketo Kato, Ignacio I. Wistuba, Anirban Maitra, Edwin J. Ostrin, Samir M. Hanash and Jody Vykoukal

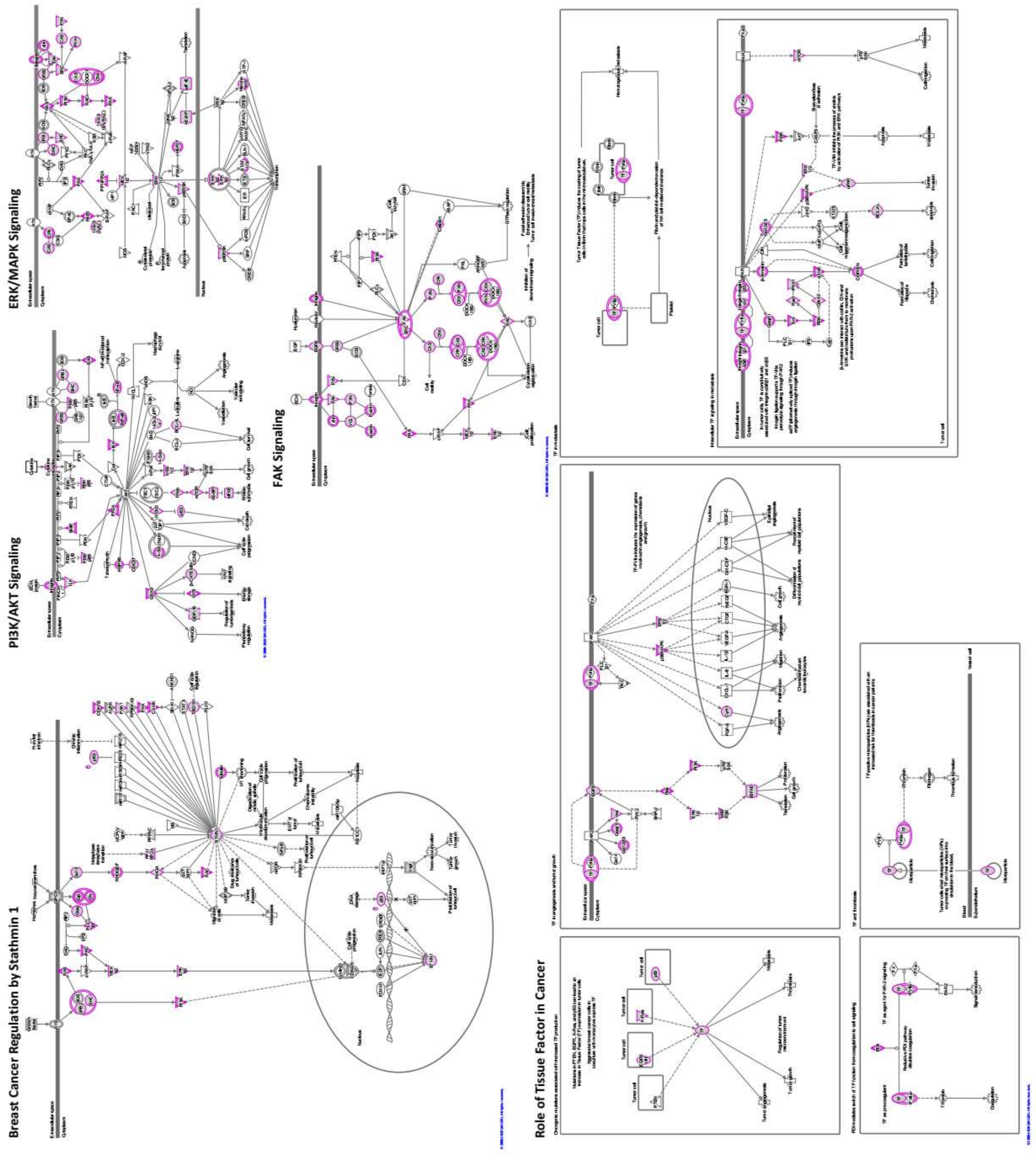

Figure S1. Ingenuity Canonical Cancer Signaling Pathways Enriched in LUAD and PDAC Cell Line sEVs.

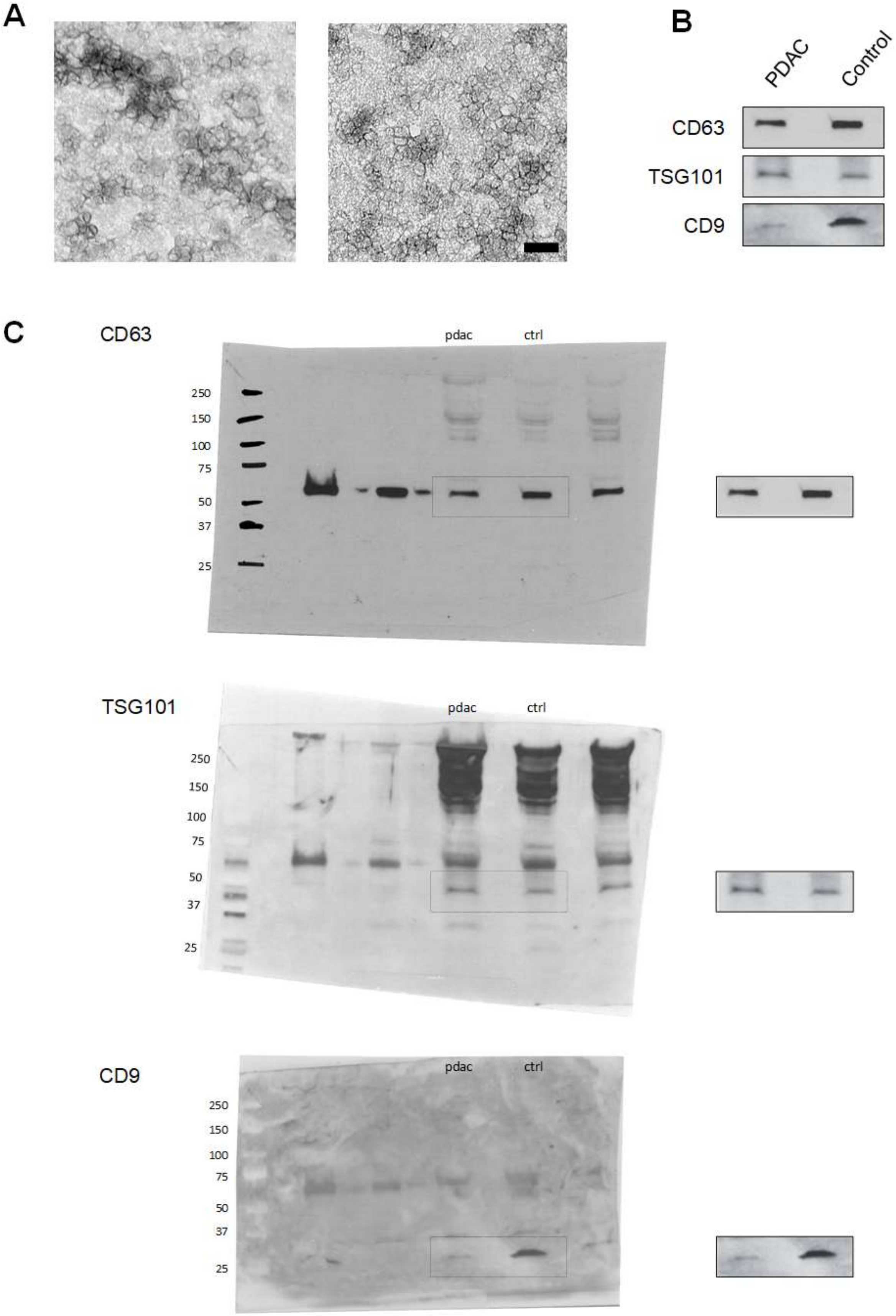

Figure S2. Characterization of plasma-derived sEVs. **(A)** Representative TEM micrograph of sEVs isolated from the plasma of a PDAC patient (left) and healthy control (right). **(B)** Western-blot analysis of expression of canonical sEV/exosome markers CD63, TSG101, and CD9 in plasma-derived sEVs. (**C**) Complete western-blots for cropped features shown in (**B**).

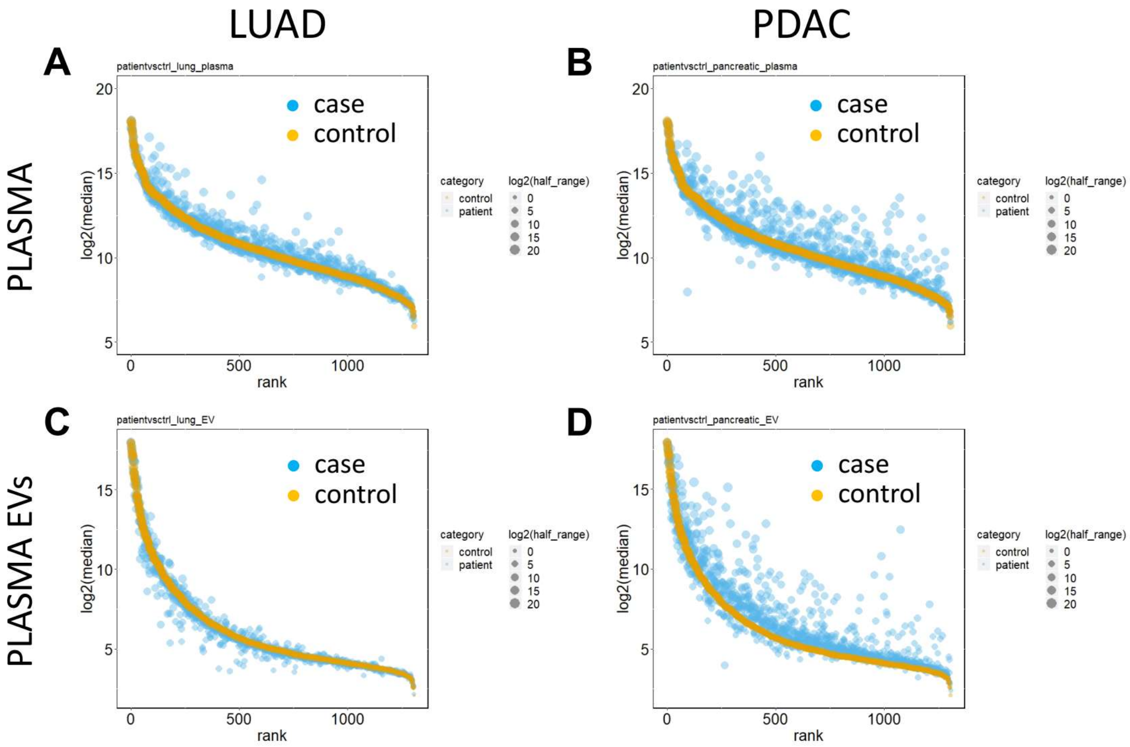

Figure S3. Distribution of proteins among LUAD and PDAC cases and controls in plasma-derived sEVs and unfractionated plasmas as analyzed by aptamer array (**A**) and (**B**) Unfractionated total plasma; (**C**) and (**D**) Plasma-derived sEVs; (**A**) and (**C**) LUAD; (**B**) and (**D**) PDAC.

© 2020 by the authors. Licensee MDPI, Basel, Switzerland. This article is an open access article distributed under the terms and conditions of the Creative Commons Attribution (CC BY) license (http://creativecommons.org/licenses/by/4.0/).
